# Risk factors for post-caesarean nausea and vomiting: a prospective prognostic study

**DOI:** 10.1016/j.bjane.2020.08.006

**Published:** 2020-09-02

**Authors:** Gabriel Magalhães Nunes Guimarães, Helga Bezerra Gomes da Silva, Hazem Adel Ashmawi

**Affiliations:** a Universidade de Brasília, Brasília, DF, Brazil; b Universidade de São Paulo, São Paulo, SP, Brazil

**Keywords:** Postoperative nausea and vomiting, Risk factors, Cesarean section, Náuseas e vômitos pós-operatórios, Fatores de risco, Parto cesariano

## Abstract

**Background:**

Postoperative Nausea and Vomiting (PONV) risk factors have not been defined for obstetric patients. In this study, our objective was to identify potential risk factors for PONV after cesarean sections performed under spinal anesthesia.

**Methods:**

One cohort of patients submitted to cesarean under spinal anesthesia was used to investigate potential risk factors for PONV. The best numerical risk factors were dichotomized using chi-squared method. A conditional independence (incremental association method) casual network was used to select the best predictors for PONV.

**Results:**

Two hundred and fifty of 260 patients remained in the study. Odds ratio for PONV of younger maternal age (< 25 years: 2.9 [1.49−5.96]), lower spinal bupivacaine dose (< 13 mg, inf [2.4-inf]), lower spinal morphine dose (< 80 mg, 0.03 [0−0.97]), history of motion sickness (2.5 [1.27−5.25]), significant nausea during the first trimester (0.3 [0.16−0.64]), intraoperative nausea and vomiting (8.2 [3.67−20.47]), and lower gestational age (< 38 weeks, 2.0 [1.01−4.08]) were statistically significant. The causal network selected absence of significant nausea during the first gestational trimester, intraoperative nausea, and gestational age < 38 weeks as the main direct risk factors for PONV.

**Conclusions:**

Intraoperative nausea and maternal age < 25 years were the main risk factors for PONV after cesareans under spinal anesthesia. Absence of self-reported nausea during the first trimester was a protective factor for post-cesarean nausea and vomiting.

## Resumo

**Introdução:**

Os fatores de risco para náusea e vômitos pós-operatórios (NVPO) ainda não foram definidos para pacientes obstétricas. Neste estudo, nosso objetivo foi identificar potenciais fatores de risco para NVPO após parto cesariano realizado sob raquianestesia.

**Método:**

Uma coorte de pacientes submetidas a cesariana sob raquianestesia foi usada para investigar potenciais fatores de risco para NVPO. Os melhores fatores de risco numéricos foram dicotomizados por meio do método qui-quadrado. Uma rede causal de independência condicional (método de associação incremental) foi usada para selecionar os melhores preditores de NVPO.

**Resultados:**

Das 260 pacientes iniciais, 250 completaram o estudo. A razão de chances para NVPO foi estatisticamente significante para menor idade materna (< 25 anos: 2,9 [1,49−5,96]), dose mais baixa de bupivacaina raquidiana (< 13 mg, inf [2,4-inf]), dose mais baixa de morfina raquidiana (< 80 mg, 0,03 [0−0,97]), histórico de enjoo de movimento (2,5 [1,27−5,25]), náuseas importantes durante o primeiro trimestre (0,3 [0,16−0,64]), náusea e vômitos intraoperatórios (8,2 [3,67−20,47]) e menor idade gestacional (< 38 semanas, 2,0 [1,01−4,08]). A rede causal selecionou ausência de náuseas significativas durante o primeiro trimestre gestacional, náusea intraoperatória e idade gestacional < 38 semanas como os principais fatores de risco diretos para NVPO.

**Conclusões:**

Náusea intraoperatória e idade materna < 25 anos foram os principais fatores de risco para NVPO após cesariana sob raquianestesia. A ausência de náusea autorreferida durante o primeiro trimestre foi um fator protetor para náusea e vômitos após cesariana.

## Introduction

Postoperative Nausea and Vomiting (PONV), although not often a threat to life, is one of the most common complications after anesthesia. PONV prophylaxis decisions must balance costs, side effects and predicted benefits. After Apfel showed that benefits are lower in low risk populations,^1^ the Society for Ambulatory Anesthesia stated in their consensus guidelines for PONV that prophylaxis should consider individual patients’ risks.^2^

Individual patient’s PONV risk is usually estimated using multivariable models. Although there are many multivariable models available for predicting PONV, Apfel’s heuristic is the most popular and used worldwide.^3^ However, Apfel’s model may lose discrimination power when one or more predictors do not vary in specific populations. One example of a population in which Apfel’s simplified model may lose discrimination power is the obstetric population as at least sex will not vary, and smoking will vary less. If we consider only the specific subgroup of cesarean patients who were submitted to spinal anesthesia, another of Apfel’s model predictors (prediction of postoperative opioid need) should not vary significantly.

PONV incidence is higher in the obstetric population and pharmacological treatment and prophylaxis’ effects to the fetus (via placental transfer) or to the newborn (during breastfeeding) are not established. Chestnut's Obstetric Anesthesia (5. ed.) cites Apfel model’s risk factors for PONV although they have not been studied specifically in obstetric patients.^4^ Our objective was to investigate risk factors for PONV in this specific population.

## Methods

This study is part of a larger project registered in ClinicalTrials NCT03171688 (posted on May 31, 2017). We planned to investigate risk factors first and then select and compare multivariable models for PONV after cesareans. After approval by the local Ethics Committee (Comitê de Ética do Hospital Oftalmológico, approval number 1.441.360), a single prospective cohort of women submitted to cesarean under spinal anesthesia was observed for 24 hours. All participants signed a written informed consent before the surgery and confirmed their willingness to participate 24 hours after. PONV occurrence (dichotomic) was the main effect studied. The main effect was true if either nausea or vomiting were present and false if both were not present. This manuscript follows TRIPOD guidelines.^5^

### Source of data

Between May 1, 2016 and June 1, 2018 we assessed all pregnant women who had a cesarean section in Hospital Universitário de Brasília, Brasilia, Brazil for eligibility in this study. The Hospital Universitário de Brasilia is a public tertiary hospital managed by the University of Brasilia. This hospital is the referral center for high-risk pregnancy (high risk for either mother or fetus) but also assists low risk pregnant women.

### Inclusion criteria

Pregnant women who had a cesarean section with spinal anesthesia and consented to participate.

### Exclusion criteria

Cesarean section did not occur;

Spinal anesthesia was not provided;

Another anesthesia technique was added to spinal.

### Other relevant sample characteristics

All women in our sample received intravenous dexamethasone 8 mg and ondansetron 8 mg during the cesarean section and were prescribed rescue intravenous metoclopramide 10 mg (institutional guideline). We also observed that no postoperative prophylactic antiemetic was prescribed to our sample. Bupivacaine and morphine doses in spinal anesthesia varied between patients because they were used in the discretion of the attending anesthesiologist.

### Outcomes

We defined PONV as either nausea, vomiting (or retching), or both nausea and vomiting up to 24 hours after the cesarean section (after skin closure). We defined Intraoperative Nausea or Vomiting (IONV) as nausea or vomiting (either or both) occurring prior to spinal anesthesia to the skin closure.

We observed IONV directly during the anesthesia. PONV was assessed retrospectively by asking participants about nausea and vomiting during the first 24 postoperative hours and by verifying nurse’s notes and the medications used during the first 24 hours. PONV was observed by anesthesiologists only during stay in the postoperative anesthesia care unit for the first two postoperative hours because there was an institutional routine of observing them for at least two hours (an obstetric team’s routine). We asked the participants and verified their records for maximal nausea intensity (verbal numerical scale 0−10) and number of vomiting episodes, but we did not take notes on the exact time nausea or vomiting occurred. The assessment of the outcome was not blinded. We stratified PONV during the first two hours (early PONV) from PONV between 2 and 24 postoperative hours (late PONV).

### Predictors

We did not include two of Apfel’s predictors, female sex, and prediction of postoperative opioid need because they were present in all patients. Before surgery, all patients were asked about motion sickness and smoking (if never smoked, if still smokes, if quit smoking less than 1 month, between 1 to 3 months, between 3 to 6 months, or more than 6 months before the surgery) because we have shown this discrimination can impact the expected probability of PONV.^6^ We also asked about significant nausea during the first trimester and during the third trimester questioning, for example, “Did you experience significant nausea during the first trimester?”. We recorded Apfel simplified model’s score for each participant.

Patients’ age and gestational age were obtained from patient records before the surgery. Mean Arterial Pressure (MAP) was measured just before spinal anesthesia and defined as basal MAP. The lowest MAP after spinal anesthesia was also recorded. Two secondary variables were recorded: absolute MAP drop (basal−lowest MAP) and relative MAP drop ([basal−lowest] divided by basal MAP). Ephedrine usage (rescue, prophylactic, or no use) was recorded as a potential predictor, but its administration was subjectively chosen by the anesthesiologist in charge of the case.

After the cesarean section, notes on spinal anesthetics were checked. Hyperbaric bupivacaine (mg), sufentanil (μg), fentanyl (μg), and morphine (μg) doses were recorded prospectively. No modeling was attempted before completion of the study cohort because we would not be able to blind the predictors to the participant assessing the outcomes.

### Sample size estimation

We initially predicted PONV incidence to be about 50% based on a previous study.^7^ Considering selecting models with up to 10 variables and the rule of thumb of 10 cases for each predictor, about 100 patients would be necessary for the model development. We predicted an excess of 30% for loss of follow up and other dropouts and another 100 patients for model validation (230 patients expected). During data collection, our data scientist warned that our PONV incidence was half the expected value, so we decided to double the modeling dataset to 260 (totaling 360 patients expected). This modeling dataset is the one used for this study.

### Statistical analysis

We tested the null hypothesis for the difference in the variables between the modeling and validation sets. The hypothesis tests methods were Fisher’s exact test, chi-squared test, unpaired Student's *t* test, or Wilcoxon-Mann-Whitney test, when appropriate. We also tested the null hypothesis for a normal distribution of all numerical variables using Shapiro-Wilk test to guide some of the analysis.

We tested the hypothesis of association of predictors and PONV using the same hypothesis test methods, but we also calculated the Bayes Factor for each association. The Bayes Factors calculated in this article compare the hypothesis of association to the null hypothesis, thus the greater the value, the greater the probability of accepting the association.

Since Bayesian causal models handle only categorical or only numerical variables, we used the chi-squared method for discretization of some potential numeric predictors (gestational age, spinal morphine dose, patient age), thus younger maternal age was defined as true if maternal age was lower than 25 years and younger gestational age was defined as true if the pregnancy length was lower than 38 weeks.

Later, a blacklist of variables (for directed conditional dependence) was determined: PONV could not be a cause of any predictor and age could not be caused by any potential predictor. Also, some variables were added to the whitelist, such as ephedrine been determined by hypotension. Finally, Incremental Association (IAMB) was used to build the acyclic graph.^8^ For better graphics, some variables were dichotomized: maternal age less than 25 years was named younger, and gestational age less than 38 weeks was named preterm. Dichotomization was performed using Chi-squared method. We used *bnlearn* package for R to model the Bayesian Network.

We calculated adjusted *p*-values using False Discovery Rate.^9^ All data analysis was performed in R Studio and a RMarkdown file was made and published in RPubs (https://rpubs.com/gabrielmng/NCT03171688). We calculated Bayes Factors with the BayesFactors package. Incremental Association (IAMB) from *bnlearn* package for R was used to model the Bayesian network.

## Results

**Figure 1:**
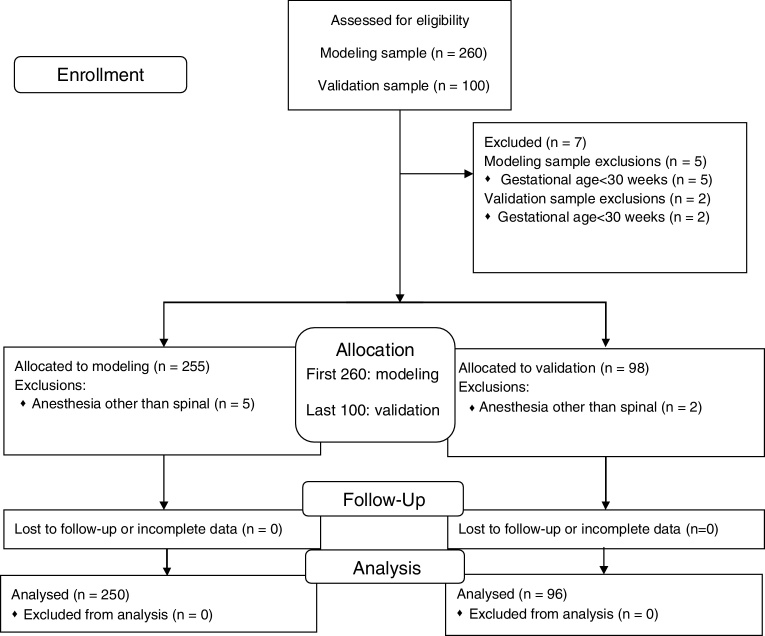
Study flow chart.

Two hundred and fifty patients remained from the 260 initial patients. Figure 1 details the inclusions and exclusions during this study. Five patients were excluded from the modeling cohort and two from the validation cohort because the gestational age was lower than 30 weeks. More detailed result data can be obtained in RPubs (http://rpubs.com/gabrielmng/NCT03171688). Since we had no missing data, no imputation method was needed. De-identified study data are available at DOI: 10.17632/p6b284247n.

PONV incidence (24 h) was lower than we expected (19.6%), with a higher proportion after discharge from PACU (Table 1). The full list of adjusted *p*-values for multiple comparisons is available in RPubs.

**Table 1:** Primary and secondary main outcomes distribution.

Characteristic	Number (%) or mean (SD)
PONV 24 h	49 (19.6%)
Max. nausea intensity 0−2 h	9.3 (1.7)
Vomiting events 0−2 h	1.6 (0.77)
Max. nausea intensity 2−24 h	4.7 (4.3)
Vomiting events 2−24 h	3 (2.2)
PONV 0−2 h	29 (11.6%)
PONV 2−24 h	20 (28.9%)
PONV 0−2 h and 2−24 h	9 (3.6%)

Data are count (proportion) or mean (SD).

We tested the null hypothesis for differences in numerical (Table 2) and dichotomic (Table 3) potential risk factors for PONV. Bayes factors were calculated to help interpretation. Only intraoperative nausea, gestational age less than 38 (preterm), and nausea during the first trimester were selected as direct PONV predictors and used in the Bayesian causal network.

**Table 2:** Analysis of potential numerical post-cesarean nausea and vomiting risk factors.

	PONV		No PONV			
	Mean	SD	Mean	SD	Raw *p*-value	Bayes Factor
Age	26.12	6.81	29.79	7.05	0.00	23.52
Gestational age	37.89	2.60	38.25	2.17	0.44	0.26
Basal MAP (mmHg)	96.40	17.64	91.27	13.90	0.15	1.55
Spinal Bupivacaine (mg)	10.73	2.04	11.73	1.42	0.04	263
Spinal Sufentanil (mcg)	1.36	1.20	1.41	1.20	0.82	0.17
Spinal Fentanyl (mcg)	10.20	16.07	8.03	12.34	0.62	0.28
Spinal Morphine (mcg)	93.06	9.61	88.05	16.27	0.04	1.2
Lower intraoperative MAP	65.12	23.62	66.97	14.53	0.30	0.21

MAP, Mean Arterial Pressure. The *p*-values are not adjusted for multiple comparisons in this table.

**Table 3:** Analysis of potential categorical risk factors.

	PONV					
Risk factor	Yes	No	RR	Odds (95%CI)	Raw *p*-value	Bayes Factor
Motion sickness	21	45	2.09	2.5 (1.27−5.25)	0.00	9.5
Nausea 1st trimester	25	154	0.41	0.3 (0.16−0.64)	0.00	67
Nausea 3rd trimester	24	75	1.46	1.6 (0.82−3.17)	0.14	0.58
IONV	40	70	5.66	8.2 (3.67−20.47)	0.00	1.1e+07
Age < 25 years	25	52	2.34	2.9 (1.49−5.96)	0.00	45
Ephedrine						
*No*	9	16	2.02	2.5 (0.9−6.7)	0.05	1.08
*Prophylactic*	12	67	0.70	0.6 (0.2−1.3)	0.30	0.35
*Rescue*	28	118	0.94	0.9 (0.4−1.8)	0.87	0.19
Gestational age < 38 weeks	21	54	1.75	2.0 (1.01−4.08)	0.03	1.8
Smoking status						
*Smokes*	0	13	0	0 (0−1.3)	0.07	0.45
*Stopped < 1 month*	0	0	NA	NA	NA	NA
*Stopped 1−3 months*	0	2	0	0 (0−21)	1	0.03
*Stopped 3−6 months*	0	0	NA	NA	NA	NA
*Stopped > 6 months*	4	28	0.58	0.5 (0.13−1.69)	0.34	0.21
*Never smoked*	43	158	2.60	3.0 (1.03−12.32)	0.04	1.5

IONV, Intraoperative Nausea and Vomiting. The *p*-values are not adjusted for multiple comparisons in this table.

## Discussion

This study has limitations and samples came from the same hospital – a public University Hospital that receives mostly poor patients; race is difficult to determine in Brazil. Our sample size is not big enough to determine a narrow sensitivity and specificity confidence interval. We had to increase our originally planned sample size because PONV incidence was much lower than expected. Also, some promising risk factors, such as spinal morphine dose, could not be predictive in our data because it presented low variation.

We observed a PONV incidence lower than Harnett and Nortcliffe^7^, ^8^, ^9^, ^10^ This lower incidence reduced the power of our sample to select multivariable models with more predictors, which may be the reason why the Bayesian Network could not include motion sickness and other important risk factors to the model.

Interestingly, the number of patients who presented nausea in the first 2 hours is the same as those who presented nausea 2−24 hours after cesarean in both datasets and many of them were the same patients. Nausea during the first two postoperative hours was associated with nausea 2−24 hours after cesarean in our sample, but most of our patients received spinal morphine, which is associated with late PONV, and spinal fentanyl or sufentanil, that has been associated with early PONV. We cannot assess the association between these opioids and PONV in this study due to its observational nature: groups would be very imbalanced (10% received no lipophilic opioid and only three patients did not receive morphine), so the study power would be low and other observational bias would be present.

We limited this study to predict dichotomic PONV up to 24 hours after cesarean, although we know that nausea intensity, the number of vomiting events, early (0−2 h) or delayed (2−24 h) PONV are important for understanding the physiopathology and to develop prophylactic strategies,^11^ thus we registered these secondary outcomes (except for outcomes longer than 24 h).

In this study, we evidenced that absence of significant nausea during the first trimester of gestation, intraoperative nausea and vomiting, and younger gestation age were the main PONV risk factors after cesarean section. Considering that both intraoperative nausea and not using ephedrine weres also associated with PONV (although ephedrine association is weak), it seems that preventing IONV by avoiding hypotension may also prevent PONV, which shall be tested in future randomized clinical trials. Lower bupivacaine doses were weakly associated with higher opioid doses in our sample and were associated with higher IONV. One hypothesis that could explain it is that our anesthesiologists used lower bupivacaine doses when they predicted a higher probability of hypotension, but this association needs further investigation.

Significant nausea during the first trimester was a PONV protective factor, although we had previously imagined it would be a risk factor. The mechanism of protection may be similar to one of the proposed mechanisms of chronic smoking for PONV, which would also explain why nausea during the third trimester was not protective (similar to what was observed in non-smokers who received postoperative nicotine patches).^12^ The exact mechanism of smoking reducing the incidence of PONV is not fully explained, however, there are several potential explanations. One popular explanation is that chronic exposure to one of the chemicals in tobacco may desensitize the patient to PONV. Being exposed to pregnancy-related hormones that induce nausea may also desensitize the patient to PONV.

Previous studies assessed risk factors for nausea during pregnancy, finding that motion sickness, non-smoking, previous nausea, higher placenta weight, younger patients, increasing gravidity, twin pregnancies, urinary tract infections, vaginal bleeding, oral herpes, African ethnicity, and a few others are risk factors for nausea during pregnancy.^13^, ^14^ Nausea in the first trimester is even associated with pregnancy outcomes.^15^ This finding must be confirmed in future studies.

We are also promoting transparency by previously publishing our observational study plan in ClinicalTrials, our analysis code in RPubs and our individual patient data in Mendeley data. This will not only increase the validity of this study but also help planning future studies and even validating new models developed by other authors, allowing our data to be used in future meta-analysis.

Bayes Factors are alternatives to classical hypothesis testing that guard for overfitting and are easier to interpret. Since there is no need for choosing an arbitrary cut-off value for Bayes Factors and they can be interpreted directly, this is why no cut-off value was defined in this study. Bayes factors also avoid the controversy about choosing prior probability distributions, as it does not depend on prior probabilities.

## Conclusions

Absence of self-reported nausea during the first gestational trimester, intraoperative nausea, and younger maternal age were the main risk factors for PONV after cesarean section under spinal anesthesia.

## Author's contribution

GMNG, study design, data collection, data analysis, writing the paper. HBGS, study design, data collection, writing the paper. HAA, study design, writing the paper, final approval of the version to be published.

All authors agree to be accountable for all aspects of the work thereby ensuring that questions related to the accuracy or integrity of any part of the work are appropriately investigated and resolved.

## Financial support

Domestic financing only.

## Conflicts of interest

The authors declare no conflicts of interest.
